# Diet quality and depression risk in a Japanese population: the Japan Public Health Center (JPHC)-based Prospective Study

**DOI:** 10.1038/s41598-019-43085-x

**Published:** 2019-05-09

**Authors:** Ryo Okubo, Yutaka J. Matsuoka, Norie Sawada, Masaru Mimura, Kayo Kurotani, Shoko Nozaki, Ryo Shikimoto, Shoichiro Tsugane

**Affiliations:** 1 Division of Health Care Research, Center for Public Health Sciences, National Cancer Center Japan, 5-1-1 Tsukiji, Chuo-ku, Tokyo, 104-0045 Japan; 2 Epidemiology and Prevention Group, Center for Public Health Sciences, National Cancer Center Japan, 5-1-1 Tsukiji, Chuo-ku, Tokyo, 104-0045 Japan; 3 Department of Neuropsychiatry, Keio University School of Medicine, Shinjuku-ku, Tokyo, 160-8582 Japan; 4 Department of Nutritional Education, National Institute of Health and Nutrition, 1-23-1 Toyama, Shinjuku-ku, Tokyo, 162-8636 Japan

**Keywords:** Nutrition, Risk factors

## Abstract

The association of overall diet quality based on the Japanese Food Guide Spinning Top with risk of depression is not known. This prospective cohort study aimed to determine whether higher adherence to the Japanese food guide reduced the risk of depression. Of 12,219 residents enrolled at baseline, we extracted 1,112 participants who completed a 5-year follow-up (1995) and participated in a mental health screening (2014–2015). Diet quality was scored based on adherence to the Japanese food guide and the ratio of white to red meat according to the Alternative Healthy Index and ranged from 0 (worst) to 80 (best). We calculated odds ratios and 95% confidence intervals for current psychiatrist-diagnosed depression per quartile of total score and of eight component scores with the lowest quartile as reference. Mean age of the participants was 73 years and 59% were women. Total diet quality score was not significantly associated with risk of depression 20 years after the baseline assessment. Among the eight components on the diet quality score, there was a significantly reduced risk for the highest quartile of the white to red meat ratio score. In conclusion, our results do not indicate that higher adherence to the Japanese food guide prevents depression.

## Introduction

Extensive research indicates that diet is one of the biggest risk factors for non-communicable diseases (NCDs)^1–5^. Therefore, for prevention of NCDs, several countries have introduced their own dietary recommendations or guidelines that consider their population’s eating habits. In Japan, the Ministry of Health, Labour, and Welfare and the Ministry of Agriculture, Forestry, and Fisheries jointly developed the Japanese Food Guide Spinning Top to emphasize the optimal balance and quantity of food in the daily Japanese diet. In 2016, Kurotani *et al*.^6^ found that a higher overall diet quality score was associated with a lower risk of mortality via a large-scale prospective cohort study (N = 79,594) assessing diet quality based on adherence to the Japanese food guide in 11 Japanese regions. For the influence of diet on depression, several mechanisms have been proposed based on animal studies and observational studies in humans, such as the gut microbiota^7^, inflammatory signaling^8^, and the hypothalamic–pituitary–adrenal (HPA) axis^9,10^. However, the association between diet quality and depression has remained unclear.

A recent cross-sectional study reported that a higher overall quality score based on adherence to the Japanese food guide was associated with lower risk of self-reported depressive symptoms in young and middle-aged women^11^. Although a recent meta-analysis of 24 independent cohorts (totaling 1,959,217 person-years) also reported that higher diet quality, regardless of type, was associated with a lower risk of depressive symptoms^12^, there was no effect of diet quality on depression in studies using a formal diagnosis as an outcome^12–15^ or when there was a control for depressive symptoms at baseline^12^. Furthermore, to our knowledge, no prospective study has examined the influence of diet quality on the risk of depression formally diagnosed by a psychiatrist.

In addition, the meta-analysis also reported the association of healthy and unhealthy food groups with the risk of depressive symptoms^12^, with significant risk reductions found for fish and vegetables. However, no study has examined the association of adherence to food groups with the risk of psychiatrist-diagnosed depression. The Japanese food guide makes recommendations on seven components: grain dishes, vegetable dishes, fish and meat dishes, milk, fruits, total energy, and snacks and alcoholic beverages. However, the Japanese food guide takes into account the total amount of fish and meat dishes but does not distinguish between the two. While higher meat consumption may increase the incidence of depression^16^, higher fish consumption may reduce its incidence^12^. Thus, to assess diet quality and consider the contents of fish and meat dishes in reference to the Alternate Healthy Eating Index^6,17^, we added the ratio of white to red meat to the seven components of the Japanese food guide.

Thus, this prospective population-based cohort study examined whether higher adherence to the Japanese food guide reduces the risk of psychiatrist-diagnosed depression. In addition, we explored the association of adherence to the recommendations for eight components—grain dishes, vegetable dishes, fish and meat dishes, milk, fruits, total energy, snacks and alcoholic beverages, and the ratio of white to red meat—with risk of depression.

## Materials and Methods

### Study population

**Figure 1:**
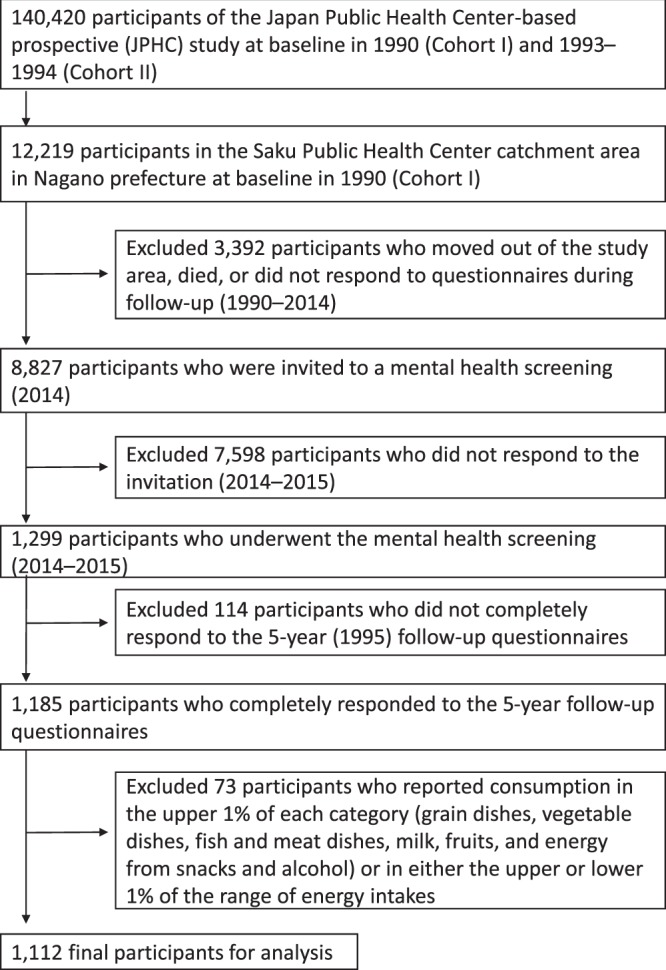
Flow diagram of study participants.

This study was conducted in one region of the Japan Public Health Center-based prospective study (JPHC study), which was started in 1990^18^. A self-administered questionnaire on demographic characteristics, past medical history, and lifestyle-related factors was distributed at baseline and at 5- and 10-year follow-ups. The flow diagram of study participants is shown in Fig. 1. Among 12,219 residents aged 40–59 years at baseline in the Saku Public Health Center catchment area in Nagano prefecture, 8,827 participants were invited to a mental health screening (2014–2015) after the exclusion of 3,392 participants who moved out of the study area, died, or did not respond to questionnaires during follow-up (1990–2014). Of the 1,299 participants who participated in the mental health screening (2014–2015), 1,185 participants completed the 5-year follow-up questionnaires (1995). Participants provided written informed consent to participate in this study. We ultimately analyzed 1,112 participants (460 men and 652 women) after excluding 73 participants who reported consumption in the upper 1% of each category (grain dishes, vegetable dishes, fish and meat dishes, milk, fruits, and energy from snacks and alcohol) or in either the upper or lower 1% of the range of energy intakes. The institutional review board at the National Cancer Center Japan and Keio University School of Medicine approved all procedures. We wrote our research protocol based on the Strengthening the Reporting of Observational Studies in Epidemiology (STROBE) statement^19^ and conducted our research by adhering to the ethical principles outlined in the Declaration of Helsinki^20^.

### Dietary assessment

We used a food frequency questionnaire at the 5-year follow-up survey (1995) to collect data on 147 food items^21^. Regarding the consumption frequency of each food item, participants chose from nine options ranging from never to seven or more times a day. Participants also classified the standard portion sizes for each food item as small (50% less than standard), medium (same as standard), and large (50% more than standard). We calculated the daily food intake by multiplying the consumption frequency by the standard portion size for each food item and assessed the daily intake of energy and nutrients using the Japanese Standard Tables of Food Composition. A previous study^22^ demonstrated the validity of the food frequency questionnaire by calculating the Spearman rank correlation coefficients between the energy-adjusted intakes determined using the food frequency questionnaire and the intakes determined using 14- or 28-day diet records. The correlation coefficients ranged from 0.13 for sake to 0.75 for coffee in men and from 0.12 for shochu to 0.80 for coffee in women. Regarding test-retest reproducibility, the study also calculated the Spearman rank correlation coefficients between the energy-adjusted intakes using two food frequency questionnaires administered at a 12-month interval. The correlation coefficients ranged from 0.21 for wine to 0.79 for rice in men and from 0.37 for sake to 0.80 for coffee in women.

To assess diet quality, we calculated the diet quality score as described previously^6^ and as shown in Supplementary Table S1. The diet quality score assessed the degree of adherence to the Japanese Food Guide Spinning Top, which is a practical and concrete food guide based on dietary guidelines for the Japanese population and was developed with the aim of preventing NCDs in the Japanese population in consideration of Japanese dietary habits. This diet quality score, defined as a modified score in the previous study^6^, has eight components—grain dishes, vegetable dishes, fish and meat dishes, milk, fruits, total energy, snacks and alcoholic beverages, and ratio of white to red meat—, with a score of 10 allocated to those who consumed the recommended amount of each component. The ratio of white to red meat was added to the original diet quality score in reference to the Alternate Healthy Eating Index^6,17^. The recommended amount of each component was specified by sex, age, and level of physical activity based on the Japanese Food Guide Spinning Top (Supplementary Table S1). When a participant exceeded or fell short of the recommended amount of each component, the score was allocated proportionately between 0 and 10 based on the degree of adherence to the recommended amount. Finally, each score was rounded off to the nearest whole number. All component scores were summed to give the total score, which ranged from 0 (lowest adherence) to 80 (highest adherence).

### Follow-up and outcome

We followed up the participants annually and determined any changes in participants using the residential registry in the Saku area until the present screening in 2014 and 2015. We identified cancer incidence using data from the major hospital in this area. Because we identified the incidence of stroke and myocardial infarction from the data of this hospital in 2009, we identified past history of depression, diabetes mellitus, stroke, and myocardial infarction by questionnaire in the present screening. Certified psychiatrists diagnosed depression based on Diagnostic and Statistical Manual of Mental Disorders, 4^th^ Edition (DSM-IV) criteria in reference to the Patient Health Questionnaire-9 (PHQ-9)^23,24^ and the Center for Epidemiological Scale-Depression (CES-D)^25,26^, which had been distributed beforehand, as described elsewhere^27^.

### Statistical analysis

Logistic regression analyses were conducted to calculate odds ratios (ORs) and 95% confidence intervals (CIs) for current depression diagnosis per quartile of the total score and of each component score on the Japanese food guide with the lowest quartile as reference. We used two models to estimate the ORs and 95% CIs. The first model was adjusted for age (years, continuous) and sex. The second model was further adjusted for living alone (yes or no), education (primary education, lower secondary education, upper secondary education, post-secondary education), smoking status (never, former, current), alcohol frequency (almost never, 1–3 times per month, ≥1 times per week), physical activity (metabolic equivalent task h/day, continuous), past history of depression (yes or no), cancer (yes or no), stroke (yes or no), myocardial infraction (yes or no), and diabetes mellitus (yes or no). We evaluated the linear trend by treating the quartile of the total score and each component score on the Japanese food guide as a continuous variable. Statistical significance was set at *p* < 0.05 (two-tailed). All analyses were performed using SAS software version 9.1 (SAS Institute, Cary, NC, USA).

### Disclaimer

The views expressed in this article are those of the authors and do not necessarily represent the views of the National Cancer Center Japan.

## Results

Mean age of the 1,112 participants included in the final analysis was 73 years and 59% were women. Median total score on the Japanese food guide was 58 (range, 25 to 80) points. As shown in Table 1, significant differences were found in sex, history of cancer and stroke, current smoking, and regular drinking between individuals with lower scores (lower quality diet) and individuals with higher scores (high quality diet). Participants with a higher quality diet tended to be female, without history of cancer and stroke, and without smoking and drinking habits. Of the 1,112 participants, 85 (7.6%) were diagnosed with depression, with mean scores of 19.3 (standard deviation = 4.5) for the CES-D and 7.1 (standard deviation = 3.3) for the PHQ-9.

**Table 1:** Participant characteristics (N = 1,112) according to the total score on the Japanese food guide (lower score = lower adherence = lower quality diet).

	Total score on the Japanese food guide				
	Lowest	Second	Third	Highest	P_diffence_
Median (min–max) score	45 (25–49)	54 (50–57)	61 (58–63)	68 (64–80)	—
*Information at screening*					
Age, years, mean ± standard deviation	72.5 ± 5.3	73.0 ± 5.8	73.3 ± 5.6	73.5 ± 5.6	0.17
Male (%)	79.3	53.6	29.2	7.7	<0.0001
History of depression (yes), %	3.9	1.0	1.9	2.4	0.15
History of diabetes (yes), %	13.3	11.0	9.7	6.7	0.07
History of cancer (yes), %	15.2	17.5	10.5	9.1	<0.01
History of stroke (yes), %	6.6	3.8	2.3	0.7	0.02
History of myocardial infraction (yes), %	2.7	2.4	2.6	0.7	0.26
*Information from baseline survey*					
Post-secondary education, %	17.2	20.6	18.4	22.3	0.31
*Information from 5-year follow-up survey*					
Living alone, %	1.2	1.7	4.1	1.3	0.06
Current smoker, %	38.6	19.6	10.9	3.7	<0.0001
Regular drinker (yes), %	71.5	44.0	31.1	19.5	<0.0001
Physical activity (METs), mean ± standard deviation	39.1 ± 10.0	37.6 ± 9.8	37.1 ± 9.4	37.3 ± 9.2	0.08

Abbreviation: MET, metabolic equivalents of task.

The influence of adherence to the Japanese food guide on depression risk is shown in Table 2. According to the total score on the Japanese food guide, there was no significant association with depression in the highest quartile for the total score (OR = 1.23, 95% CI = 0.58–2.63), rather also after adjustment for possible confounding factors. Although we conducted sensitivity analyses of the participants according to sex, history of depression, history of illness, current smoking, regular drinking habits, living alone, education, and physical activity, there was no significant association with depression (Table 2).

**Table 2:** Odds ratios and 95% confidence intervals for depression according to the quartile of the total score on the Japanese food guide (lower score = lower adherence = lower quality diet).

	Lowest	Second	Third	Highest	P_trend_
*Total score*					
Median (min–max) score	45 (25–49)	54 (50–57)	61 (58–63)	68 (64–80)	**—**
No. of cases/controls	19/237	19/272	19/248	28/270	**—**
Age, sex-adjusted OR (95% CI)	1.00	0.82 (0.42–1.62)	0.87 (0.42–1.79)	1.15 (0.55–2.39)	0.61
Multivariate OR^a^ (95% CI)	1.00	0.91 (0.45–1.83)	0.96 (0.45–1.95)	1.23 (0.58–2.63)	0.52
*Male (n = 460)*					
Median (min–max) score	41 (25–44)	47 (47–50)	53 (51–56)	61 (57–75)	**—**
No. of cases/controls	9/96	8/108	6/116	9/108	**—**
Age-adjusted OR (95% CI)	1.00	0.82 (0.30–2.24)	0.57 (0.19–1.65)	0.93 (0.35–2.49)	0.74
Multivariate OR^a^ (95% CI)	1.00	0.78 (0.28–2.20)	0.58 (0.19–1.80)	1.10 (0.38–3.16)	0.99
*Female (n = 652)*					
Median (min–max) score	52 (39–55)	59 (56–61)	64 (62–61)	71 (67–80)	**—**
No. of cases/controls	8/135	15/148	16/157	14/159	**—**
Age-adjusted OR (95% CI)	1.00	1.58 (0.64–3.88)	1.69 (0.70–4.09)	1.30 (0.53–3.24)	0.65
Multivariate OR^a^ (95% CI)	1.00	1.46 (0.58–3.67)	1.77 (0.71–4.41)	1.16 (0.45–2.97)	0.78
*No past history of depression (n = 1087)*					
Median (min–max) score	45 (25–49)	54 (50–57)	61 (58–63)	68 (64–80)	**—**
No. of cases/controls	17/229	18/270	19/243	26/265	**—**
Age, sex-adjusted OR (95% CI)	1.00	0.85 (0.42–1.72)	0.97 (0.46–2.02)	1.18 (0.56–2.51)	0.55
Multivariate OR^a^ (95% CI)	1.00	0.87 (0.42–1.79)	1.00 (0.47–2.13)	1.22 (0.56–2.68)	0.50
*No past history of illness (n = 817)*					
Median (min–max) score	45 (25–50)	55 (51–57)	61 (58–64)	68 (65–78)	**—**
No. of cases/controls	13/176	11/178	16/207	20/196	**—**
Age, sex-adjusted OR (95% CI)	1.00	0.84 (0.36–1.97)	1.06 (0.47–2.42)	1.38 (0.58–3.28)	0.36
Multivariate OR^a^ (95% CI)	1.00	0.91 (0.38–2.18)	1.17 (0.50–2.73)	1.47 (0.60–3.61)	0.30
*Not current smoker (n = 917)*					
Median (min–max) score	47 (28–51)	55 (52–58)	62 (59–64)	69 (65–80)	**—**
No. of cases/controls	14/183	21/217	11/210	27/234	**—**
Age, sex-adjusted OR (95% CI)	1.00	1.19 (0.58–2.47)	0.64 (0.27–1.52)	1.32 (0.61–2.87)	0.68
Multivariate OR^a^ (95% CI)	1.00	1.25 (0.60–2.62)	0.69 (0.29–1.66)	1.42 (0.64–3.11)	0.56
*Not regular drinker (n = 660)*					
Median (min–max) score	49 (33–53)	57 (54–60)	63 (61–65)	70 (66–80)	**—**
No. of cases/controls	7/132	17/169	10/138	16/171	**—**
Age, sex-adjusted OR (95% CI)	1.00	1.66 (0.66–4.19)	1.20 (0.43–3.35)	1.40 (0.54–3.68)	0.79
Multivariate OR^a^ (95% CI)	1.00	1.59 (0.62–4.06)	1.23 (0.43–3.46)	1.37 (0.52–3.62)	0.80
*Not living alone (n = 1089)*					
Median (min–max) score	45 (25–49)	54 (50–57)	61 (58–63)	68 (64–80)	**—**
No. of cases/controls	19/234	19/267	18/238	27/267	**—**
Age, sex-adjusted OR (95% CI)	1.00	0.83 (0.42–1.64)	0.86 (0.42–1.78)	1.12 (0.54–2.33)	0.68
Multivariate OR^a^ (95% CI)	1.00	0.86 (0.43–1.72)	0.91 (0.44–1.91)	1.19 (0.56–2.52)	0.56
*Not post-secondary education (n = 915)*					
Median (min–max) score	45 (25–49)	54 (50–57)	61 (58–63)	68 (64–80)	**—**
No. of cases/controls	14/198	15/216	16/202	22/232	**—**
Age, sex-adjusted OR (95% CI)	1.00	0.84 (0.38–1.85)	0.87 (0.38–1.99)	0.99 (0.43–2.28)	0.92
Multivariate OR^a^ (95% CI)	1.00	0.87 (0.39–1.94)	0.93 (0.40–2.16)	1.04 (0.44–2.42)	0.83
*Not sedentary (n = 1010)*					
Median (min–max) score	45 (25–49)	54 (50–57)	61 (58–63)	68 (64–80)	**—**
No. of cases/controls	17/222	19/239	18/223	27/245	**—**
Age, sex-adjusted OR (95% CI)	1.00	0.99 (0.49–1.98)	0.97 (0.46–2.05)	1.29 (0.60–2.77)	0.50
Multivariate OR^a^ (95% CI)	1.00	1.01 (0.50–2.06)	1.01 (0.47–2.17)	1.36 (0.62–2.96)	0.42

^a^Adjusted for age, sex, living alone, education, smoking status, alcohol frequency, physical activity, past history of depression, cancer, stroke, myocardial infarction, and diabetes mellitus.

Among the eight components on the food quality score, there was a significantly reduced depression risk for the highest quartile of the white to red meat ratio score (OR = 0.52, 95% CI = 0.27–0.98) and a significant linear trend (*p* < 0.05) after adjustment for possible confounding factors (Supplementary Table S2). There were no significant associations of the scores with grain dishes, vegetable dishes, fish and meat dishes, milk, fruits, total energy, and snacks and alcoholic beverages with depression.

## Discussion

The aim of this study was to assess the influence of adherence to the Japanese food guide on the risk of depression. Our results showed that a higher total diet quality score (higher quality diet) was not significantly associated with a lower risk of depression. To our knowledge, this is the first prospective study to examine the association of quality of diet based on adherence to the Japanese food guide with risk of psychiatrist-diagnosed depression. Our findings also suggest that a higher ratio of white to red meat could be associated with lower risk of depression.

Rather also after adjustment for possible confounding variables, a higher total score (higher quality diet) was not significantly associated with a lower risk of psychiatrist-diagnosed depression. This result is in line with the conclusion of a recent meta-analysis that reported that the associations determined for higher diet quality differed between the risk of depressive symptoms (OR = 0.77, 95% CI = 0.69–0.84) and the risk of formally diagnosed depression (OR = 0.91, 95% CI = 0.68–1.23)^12^. This difference could be explained by the beneficial effects of a higher diet quality on NCDs. A higher quality diet is associated with a significantly reduced risk of NCDs such as cardiovascular disease, diabetes, and cancer^6,28^. Patients with these NCDs tend to have somatic symptoms such as fatigue, loss of appetite, insomnia, and work difficulty in conjunction with depression^29–31^. These somatic symptoms related to NCDs might be present regardless of the lack of core depressive symptoms such as depressed mood and anhedonia, which are required for the diagnosis of depression^32^. Hence, a self-report questionnaire could overestimate depression among patients with an NCD. Furthermore, a higher quality diet has been associated with a reduced risk of these somatic symptoms among patients with NCDs^33,34^. Therefore, a higher quality diet might not reduce the risk of a depression diagnosis but might reduce the risk of a higher score on the self-report questionnaire.

In addition, our finding that a higher quality diet was not significantly associated with a lower risk of psychiatrist-diagnosed depression could be related to differences in study design. Among the three studies that used a formal diagnosis as an outcome in the previous meta-analysis of prospective studies^12^, one study involving male and female university students found a significant relationship of the diets conforming to the Pro-vegetarian Dietary Pattern and the Alternate Healthy Eating Index 2010 with the incidence of depression (OR = 0.58, 95% CI = 0.44–0.77) in a 4-year follow-up^13^. In contrast, one study of boys and girls aged 10–11 years did not find a significant relationship between diet quality (evaluated using the Diet Quality Index-International) and the incidence of internalizing disorder including depressive and anxiety symptoms (OR = 1.09, 95% CI = 0.73–1.63) in a 4-year follow-up^15^. Finally, one study involving middle-aged and older women did not find a significant relationship between prudent dietary patterns (high in vegetables, fruit, whole grain, and fish) and the incidence of depression (OR = 1.05, 95% CI = 0.91–1.20) in a 12-year follow-up^14^, which is in line with our findings for older men and women in a 19-year follow-up. These inconsistent results could be attributable to differences in the age of the study population, dietary assessment, and follow-up period. Further studies are necessary to examine the influence of diet quality on formally diagnosed depression and to consider differences in the age of the study population, diet assessment, and follow-up period.

A recent meta-analysis including 16 randomized controlled trials found positive effects of dietary improvement on depressive symptoms (Hedges *g* = 0.275, 95%CI = 0.10–0.45 for the overall effect size of dietary interventions)^35^. However, some researchers have questioned the remarkably large effect of one of the trials. Regarding the SMILES study^36^, Molendijk *et al*.^37^ have noted the possibility that selectively induced expectancy and loss of blinding may have led to the large effect (*d* = 1.98 for the pre–post effect). Including our results showing no association between diet quality and psychiatrist-diagnosed depression, the evidence regarding the association of diet with depression is inconsistent. Therefore, to develop effective nutritional prevention of depression, it is necessary to further examine the association between diet and depression.

Our results also indicate that a higher ratio of white to red meat could be associated with a dose-dependent lower risk of depression. Compared with participants in the lowest quartile, who had an approximately equal ratio of white to red meat, the participants in the highest quartile, who had an approximately 4:1 ratio of white to red meat, had greater than a 40% reduced risk of depression. To our knowledge, this study is the first to show a relationship between the ratio of white to red meat and depression. The participants in this study consumed 90% of white meat from fish (data not shown). Our previous study has shown the beneficial effects of fish consumption on depression^27^. Thus, we assumed that fish consumption influenced our current findings on the relationship between the ratio of white to red meat and depression. Furthermore, a meta-analysis of three cohort studies showed that higher meat consumption was associated with a higher incidence of depression (relative risk = 1.13, 95% CI = 1.03–1.24)^16^. These previous studies indicated that depression could be affected by the balance between the consumption of white meat, including fish, and red meat. The balance between the consumption of white meat and red meat could be an additional component of the Japanese food guide and its effects on depression should be examined in further prospective studies.

Although the strengths of this study are its prospective design, controls for possible confounding, and the psychiatrist-certified diagnosis of depression, we need to mention several limitations. First, because it was necessary for the participants in this study to come to a specific place on a specific day when the certified psychiatrist was available, only 14% of the residents enrolled at baseline (1990) participated in this study (2014–2015). Their participation could also be based on their health-related preferences or motivations, and thus might cause selection bias. Second, we unfortunately did not have baseline information on depression. The aforementioned systematic review^12^ reported that there is no effect of diet quality on depression when there is a statistical control for baseline depressive symptoms. Further studies should examine the influence of diet quality on psychiatrist-diagnosed depression after excluding the influence of baseline depressive symptoms. Third, we calculated diet quality at only a 5-year follow-up survey and did not assess any further changes in diet. Thus, the quality of the diet might not reflect habitual diet quality beyond the 5-year follow-up survey. However, a cohort study assessing changes in dietary patterns among older adults showed that approximately 60% of participants had a stable dietary pattern during a 10-year follow-up^38^. Therefore, we considered the diet quality at the 5-year follow-up survey to represent habitual diet quality after the 5-year follow-up survey. Finally, we did not assess depression during the follow-up period and conducted the psychiatric interview once 25 years after the baseline assessment. Although certified psychiatrists diagnosed depression based on DSM-IV criteria in reference to the PHQ-9 and CES-D, we did not use a structured clinical interview for the psychiatric diagnosis.

In conclusion, our results do not indicate that higher adherence to the Japanese food guide prevents psychiatrist-diagnosed depression. Further studies are necessary to examine the influence of diet quality on psychiatrist-diagnosed depression and to consider differences in the age of the study population, diet assessment, and follow-up period.

## Supplementary information

Supplementary
