# Supplementary material for: Diet quality and depression risk in a Japanese population: the Japan Public Health Center (JPHC)-based Prospective Study
Source: Sci Rep. 2019 May 9;9:7150. doi: 10.1038/s41598-019-43085-x (PMC6509323; doi:10.1038/s41598-019-43085-x)
Supplement: Supplementary file 1 — Supplementary [file 41598_2019_43085_MOESM1_ESM.docx]

Supplementary

**Title:**

Diet quality and depression risk in Japan: a population-based prospective cohort study

**Authors and affiliations:**

Ryo Okubo^1)^, Yutaka J Matsuoka^1)^*, Norie Sawada^2)^*, Masaru Mimura^3)^, Kayo Kurotani^4)^, Shoko Nozaki^3)^, Ryo Shikimoto^3)^, Shoichiro Tsugane^2)^

1) Division of Health Care Research, Center for Public Health Sciences, National Cancer Center Japan, 5-1-1 Tsukiji, Chuo-ku, Tokyo 104-0045, Japan

2) Epidemiology and Prevention Group, Center for Public Health Sciences, National Cancer Center Japan, 5-1-1 Tsukiji, Chuo-ku, Tokyo 104-0045, Japan

3) Department of Neuropsychiatry, Keio University School of Medicine, Shinjuku-ku,　Tokyo 160-8582, Japan

4) Department of Nutritional Education, National Institute of Health and Nutrition, 1-23-1 Toyama, Shinjuku-ku, Tokyo 162-8636, Japan

* **Corresponding authors:**

Yutaka J. Matsuoka, MD, PhD, Division of Health Care Research, Center for Public Health Sciences, National Cancer Center Japan, 5-1-1 Tsukiji, Chuo-ku, Tokyo 104-0045, Japan

Tel.: +81-3-3542-2511 ext. 3382; E-mail: yumatsuo@ncc.go.jp

Norie Sawada, MD, PhD, Epidemiology and Prevention Group, Center for Public Health Sciences, National Cancer Center Japan, 5-1-1 Tsukiji, Chuo-ku, Tokyo 104-0045, Japan

Tel.: +81-3-3547-5201 ext. 3336; E-mail: nsawada@ncc.go.jp

Drs. Yutaka J. Matsuoka and Norie Sawada contributed equally as corresponding authors.

Supplementary Table S1. Methods for calculating diet quality score based on the Japanese food guide

|  | Recommended amount by the Japanese food guide | Standard for a minimum score of 0 points | Standard for a continuous score of 0–10 points^§^ | Standard for a maximum score of 10 points |
| --- | --- | --- | --- | --- |
| ***Grain dishes*^£^** |  |  |  |  |
| Men aged ≥70; women aged ≥70 or sedentary women aged 18–69 (serving/d) | 4–5 | 0 or ≥10 | 0–4 or 5–10 | 4–5 |
| Sedentary men aged 18–69; moderately active women aged 18–69 (serving/d) | 5–7 | 0 or ≥14 | 0–5 or 7–14 | 5–7 |
| Moderately active men aged 18–69 (serving/d) | 7–8 | 0 or ≥16 | 0–7 or 8–16 | 7–8 |
| ***Vegetable dishes*^*,^**^¶^ |  |  |  |  |
| Men aged ≥70; women aged ≥70 or sedentary women aged 18–69 (serving/d) | 5–6 | 0 | 0–5 | ≥5 |
| Sedentary men aged 18–69; moderately active women aged 18–69 (serving/d) | 5–6 | 0 | 0–5 | ≥5 |
| Moderately active men aged 18–69 (serving/d) | 6–7 | 0 | 0–5 | ≥6 |
| ***Fish and meat dishes***^Π^ |  |  |  |  |
| Men aged ≥70; women aged ≥70 or sedentary women aged 18–69 (serving/d) | 3–4 | 0 or ≥8 | 0–3 or 4–8 | 3–4 |
| Sedentary men aged 18–69; moderately active women aged 18–69 (serving/d) | 3–5 | 0 or ≥10 | 0–3 or 5–10 | 3–5 |
| Moderately active men aged 18–69 (serving/d) | 4–6 | 0 or ≥12 | 0–4 or 6–12 | 4–6 |
| ***Milk***^Φ^ |  |  |  |  |
| Men aged ≥70; women aged ≥70 or sedentary women aged 18–69 (serving/d) | 2 | 0 or ≥4 | 0–2 or 2–4 | 2 |
| Sedentary men aged 18–69; moderately active women aged 18–69 (serving/d) | 2 | 0 or ≥4 | 0–2 or 2–4 | 2 |
| Moderately active men aged 18–69 (serving/d) | 2–3 | 0 or ≥6 | 0–2 or 3–6 | 2–3 |
| ***Fruits^*,^***^Ω^ |  |  |  |  |
| Men aged ≥70; women aged ≥70 or sedentary women aged 18–69 (serving/d) | 2 | 0 | 0–2 | ≥2 |
| Sedentary men aged 18–69; moderately active women aged 18–69 (serving/d) | 2 | 0 | 0–2 | ≥2 |
| Moderately active men aged 18–69 (serving/d) | 2–3 | 0 | 0–2 | ≥2 |
| ***Total energy*** |  |  |  |  |
| Men aged ≥70; women aged ≥70 or sedentary women aged 18–69 (kcal/d) | 1600–2000 | ≥4000 | <1600 or 2000–4000 | 1600–2000 |
| Sedentary men aged 18–69; moderately active women aged 18–69 (kcal/d) | 2000–2400 | ≥4800 | <2000 or 2400–4800 | 2000–2400 |
| Moderately active men aged 18–69 (kcal/d) | 2400–2800 | ≥5600 | <2400 or 2800–5600 | 2400–2800 |
| ***Snacks and alcoholic beverages*** |  |  |  |  |
| Men aged ≥70; women aged ≥70 or sedentary women aged 18–69 (kcal/d) | 0–200 | ≥400 | 200–400 | 0–200 |
| Sedentary men aged 18–69; moderately active women aged 18–69 (kcal/d) | 0–200 | ≥400 | 200–400 | 0–200 |
| Moderately active men aged 18–69 (kcal/d) | 0–200 | ≥400 | 200–400 | 0–200 |
| ***Ratio of white to red meat*** |  |  |  |  |
| Men aged ≥70; women aged ≥70 or sedentary women aged 18–69 (serving/d) | 4:1 | 0 | 0–4:1 | ≥4:1 |
| Sedentary men aged 18–69; moderately active women aged 18–69 (serving/d) | 4:1 | 0 | 0–4:1 | ≥4:1 |
| Moderately active men aged 18–69 (serving/d) | 4:1 | 0 | 0–4:1 | ≥4:1 |

**^§^** If an individual consumed less than the recommended amount of servings or energy, the score was calculated with the formula: 10 × (consumed amount of servings or energy)/(lower limit of the recommended amount). If an individual consumed more than the recommended amount of servings or energy, the score was calculated with the formula: 10 – 10 × [(consumed amount of servings or energy) – (upper limit of the recommended amount)]/(upper limit of the recommended amount).

**^£^** Includes rice, bread, and noodles. One serving contains about 40 g carbohydrate.

* Recommended servings of vegetable dishes and fruits in the Japanese Food guide Spinning Top modified from equal to recommended equivalents to greater than or equal to recommended.

**^¶^** Includes vegetables, mushrooms, potatoes, and seaweed. In one serving, the main ingredient weighs about 70 g.

**^Π^** Includes meat, fish, eggs, and soybeans. One serving contains about 6 g protein.

**^Φ^** Includes milk and milk products. One serving contains about 100 mg calcium.

**^Ω^** In one serving, the main ingredient weighs about 100 g.

Supplementary Table S2. Odds ratios and 95% confidence intervals for depression according to the quartile of the total score and each component score on the Japanese food guide (lower score = lower adherence = lower quality diet)

|  | Lowest | Second | Third | Highest | P_trend_ |
| --- | --- | --- | --- | --- | --- |
| *Grain dishes* |  |  |  |  |  |
| Median (min–max) score | 6 (2–6) | 7 (7–7) | 8 (8–8) | 9 (9–10) | **-** |
| No. of cases/controls | 20/249 | 11/144 | 17/212 | 37/422 | **-** |
| Age, sex-adjusted OR (95% CI) | 1.00 | 0.91 (0.42–1.95) | 0.96 (0.48–1.90) | 1.03 (0.58–1.84) | 0.86 |
| Multivariate OR^a^ (95% CI) | 1.00 | 0.99 (0.45–2.15) | 1.07 (0.53–2.15) | 1.14 (0.63–2.06) | 0.62 |
| *Vegetable dishes* |  |  |  |  |  |
| Median (min–max) score | 4 (1–5) | 6 (6–7) | 9 (8–9) | 10 (10–10) | **-** |
| No. of cases/controls | 14/227 | 14/197 | 21/211 | 36/392 | **-** |
| Age, sex-adjusted OR (95% CI) | 1.00 | 1.12 (0.52–2.40) | 1.49 (0.72–3.01) | 1.31 (0.67–2.56) | 0.39 |
| Multivariate OR^a^ (95% CI) | 1.00 | 1.02 (0.46–2.24) | 1.41 (0.67–2.96) | 1.30 (0.65–2.61) | 0.37 |
| *Fish and meat dishes* |  |  |  |  |  |
| Median (min–max) score | 0 (0–4) | 6 (5–7) | 9 (8–9) | 10 (10–10) | **-** |
| No. of cases/controls | 17/225 | 21/194 | 14/262 | 33/346 | **-** |
| Age, sex-adjusted OR (95% CI) | 1.00 | 1.52 (0.78–2.99) | 0.78 (0.37–1.64) | 1.38 (0.74–2.57) | 0.59 |
| Multivariate OR^a^ (95% CI) | 1.00 | 1.47 (0.74–2.92) | 0.77 (0.36–1.62) | 1.40 (0.75–2.63) | 0.56 |
| *Milk* |  |  |  |  |  |
| Median (min–max) score | 0 (0–0) | 2 (1–4) | 7 (5–8) | 10 (9–10) | **-** |
| No. of cases/controls | 22/312 | 12/179 | 21/232 | 30/304 | **-** |
| Age, sex-adjusted OR (95% CI) | 1.00 | 1.00 (0.47–2.12) | 1.30 (0.67–2.52) | 1.41 (0.72–2.74) | 0.25 |
| Multivariate OR^a^ (95% CI) | 1.00 | 0.94 (0.43–2.03) | 1.28 (0.64–2.53) | 1.34 (0.68–2.66) | 0.31 |
| *Fruits* |  |  |  |  |  |
| Median (min–max) score | 4 (0–5) | 7 (6–8) | 9 (9–9) | 10 (10–10) | **-** |
| No. of cases/controls | 18/235 | 19/201 | 7/81 | 41/510 | **-** |
| Age, sex-adjusted OR (95% CI) | 1.00 | 1.17 (0.59–2.31) | 1.02 (0.40–2.55) | 0.94 (0.52–1.71) | 0.66 |
| Multivariate OR^a^ (95% CI) | 1.00 | 1.27 (0.63–2.54) | 1.00 (0.39–2.55) | 1.01 (0.55–1.86) | 0.81 |
| *Total energy* |  |  |  |  |  |
| Median (min–max) score | 7 (0–7) | 8 (8–8) | 9 (9–9) | 10 (10–10) | **-** |
| No. of cases/controls | 20/215 | 16/175 | 28/386 | 21/251 | **-** |
| Age, sex-adjusted OR (95% CI) | 1.00 | 0.96 (0.48–1.92) | 0.79 (0.43–1.44) | 0.91 (0.48–1.74) | 0.64 |
| Multivariate OR^a^ (95% CI) | 1.00 | 0.93 (0.46–1.89) | 0.75 (0.40–1.38) | 0.85 (0.44–1.64) | 0.49 |
| *Snacks and alcoholic beverages* |  |  |  |  |  |
| Median (min–max) score | 0 (0–5) | 7 (6–8) | 9 (9–9) | 10 (10–10) | **-** |
| No. of cases/controls | 22/247 | 8/80 | 2/45 | 53/655 | **-** |
| Age, sex-adjusted OR (95% CI) | 1.00 | 1.03 (0.43–2.45) | 0.41 (0.09–1.82) | 0.70 (0.37–1.31) | 0.21 |
| Multivariate OR^a^ (95% CI) | 1.00 | 1.06 (0.43–2.60) | 0.42 (0.09–1.91) | 0.69 (0.35–1.39) | 0.22 |
| *Ratio of white to red meat* |  |  |  |  |  |
| Median (min–max) score | 3 (0–3) | 4 (4–4) | 6 (5–8) | 10 (9–10) | **-** |
| No. of cases/controls | 27/244 | 11/141 | 29/377 | 18/265 | **-** |
| Age, sex-adjusted OR (95% CI) | 1.00 | 0.69 (0.33–1.43) | 0.67 (0.39–1.17) | 0.56 (0.30–1.06) | 0.07 |
| Multivariate OR^a^ (95% CI) | 1.00 | 0.65 (0.31–1.38) | 0.66 (0.37–1.15) | **0.52 (0.27–0.98)** | **<0.05** |

^a^Adjusted for age, sex, living alone, education, smoking status, alcohol frequency, physical activity, past history of depression, cancer, stroke, myocardial infarction, and diabetes mellitus.
